# Increased Angiogenesis and Improved Left Ventricular Function after Transplantation of Myoblasts Lacking the *MyoD* Gene into Infarcted Myocardium

**DOI:** 10.1371/journal.pone.0041736

**Published:** 2012-07-25

**Authors:** Yasuhiro Nakamura, Yoko Asakura, Bryan A. Piras, Hiroyuki Hirai, Christopher T. Tastad, Mayank Verma, Amanda J. Christ, Jianyi Zhang, Takanori Yamazaki, Minoru Yoshiyama, Atsushi Asakura

**Affiliations:** 1 Cardiovascular Division Department of Medicine, University of Minnesota Medical School, Minneapolis, Minnesota, United States of America; 2 Department of Internal Medicine and Cardiology, Osaka City University Medical School, Osaka, Japan; 3 Stem Cell Institute, University of Minnesota Medical School, Minneapolis, Minnesota, United States of America; 4 Paul and Sheila Wellstone Muscular Dystrophy Center, University of Minnesota Medical School, Minneapolis, Minnesota, United States of America; 5 Department of Neurology, University of Minnesota Medical School, Minneapolis, Minnesota, United States of America; Clinica Universidad de Navarra, Spain

## Abstract

Skeletal myoblast transplantation has therapeutic potential for repairing damaged heart. However, the optimal conditions for this transplantation are still unclear. Recently, we demonstrated that satellite cell-derived myoblasts lacking the *MyoD* gene (*MyoD^−/−^*), a master transcription factor for skeletal muscle myogenesis, display increased survival and engraftment compared to wild-type controls following transplantation into murine skeletal muscle. In this study, we compare cell survival between wild-type and *MyoD^−/−^* myoblasts after transplantation into infarcted heart. We demonstrate that *MyoD^−/−^* myoblasts display greater resistance to hypoxia, engraft with higher efficacy, and show a larger improvement in ejection fraction than wild-type controls. Following transplantation, the majority of *MyoD^−/−^* and wild-type myoblasts form skeletal muscle fibers while cardiomyocytes do not. Importantly, the transplantation of *MyoD^−/−^* myoblasts induces a high degree of angiogenesis in the area of injury. DNA microarray data demonstrate that paracrine angiogenic factors, such as stromal cell-derived factor-1 (SDF-1) and placental growth factor (PlGF), are up-regulated in *MyoD^−/−^* myoblasts. In addition, over-expression and gene knockdown experiments demonstrate that MyoD negatively regulates gene expression of these angiogenic factors. These results indicate that *MyoD^−/−^* myoblasts impart beneficial effects after transplantation into an infarcted heart, potentially due to the secretion of paracrine angiogenic factors and enhanced angiogenesis in the area of injury. Therefore, our data provide evidence that a genetically engineered myoblast cell type with suppressed MyoD function is useful for therapeutic stem cell transplantation.

## Introduction

Stem cells have extensive proliferative potential and differentiate into several cell lineages. Therefore, stem cell transplantation remains an attractive approach for myocardial repair. Numerous cell types may ameliorate the symptoms of myocardial infarction (MI), including circulating endothelial progenitor cells (cEPCs) [1], mesenchymal stem cells (MSCs) [2], multipotent adult progenitor cells (MAPCs) [3], embryonic stem (ES) cells [4], induced pluripotent stem (iPS) cells [5], cardiac progenitor cells [6], [7], [8], vessel associated mesoangioblasts [9], [10], skeletal muscle-derived stem cells [11], and skeletal muscle myoblasts [12]. The transplantation of skeletal muscle myoblasts has been used both experimentally and clinically in an attempt to restore cardiac function [13], [14], [15], [16], [17]. Advantages to this approach include a readily available cell source and the biochemical and functional similarities between skeletal and cardiac muscle [18]. Although engrafted myoblasts improve post-infarct cardiac function [15], they differentiate into mature skeletal muscle fibers and do not appear to express cardiac-specific proteins [12], [19]. In a recent case study, myoblast transplantation improved cardiac function and mitigated symptoms, but some patients sustained episodes of ventricular tachycardia and required implantable cardioverter-defibrillators [16], [20]. Recent work suggests that stem cell transplantation can repair heart function through induction of paracrine factors that recruit hematopoietic cells [21] and induce angiogenesis and cardiomyocyte contractility in the injured heart [12], [22], [23], [24]. Hence, the production of new cardiomyocytes and vasculature by means of stem cell transplantation is an attractive approach to heart therapy.

The myogenic regulatory factors are a group of skeletal muscle-specific basic helix-loop-helix (bHLH) transcription factors, including MyoD, Myf5, myogenin, and MRF4, that play an essential role in satellite cell activation, proliferation and differentiation [25], [26]. Satellite cell-derived myoblasts lacking the *MyoD* gene (*MyoD^−/−^*) display accelerated growth rates as well as delayed terminal differentiation and muscle regeneration [27]. In addition, *MyoD^−/−^* myoblasts display more primitive characteristics than wild-type cells and represent an intermediate stage between stem cells and myogenic precursors [27], [28]. Recently, we demonstrated that *MyoD^−/−^* myoblasts engraft with significantly higher efficiency compared to wild-type myoblasts after injection into injured skeletal muscle [29]. Importantly, many anti-apoptotic genes are up-regulated in the *MyoD^−/−^* myoblast population, while genes known to promote apoptosis are down-regulated. Consistent with this gene expression profile, *MyoD^−/−^* myoblasts display remarkable resistance to apoptosis and increased cell survival [30], [31]. Therefore, *MyoD^−/−^* myoblasts may be useful for the treatment of damaged heart tissue.

In this study, we investigate whether (1) *MyoD^−/−^* myoblasts display significantly higher engraftment in infarcted mouse heart compared to wild-type myoblasts; (2) engrafted *MyoD^−/−^* myoblasts improve cardiac function in the infarcted heart; (3) *MyoD^−/−^* myoblasts can differentiate into cardiomyocytes; and (4) *MyoD^−/−^* myoblasts can induce angiogenesis in the injured area of the heart.

## Results

### Isolation of Wild-type and *MyoD^−/−^* Myoblasts for Cardiac Repair

**Figure 1:**
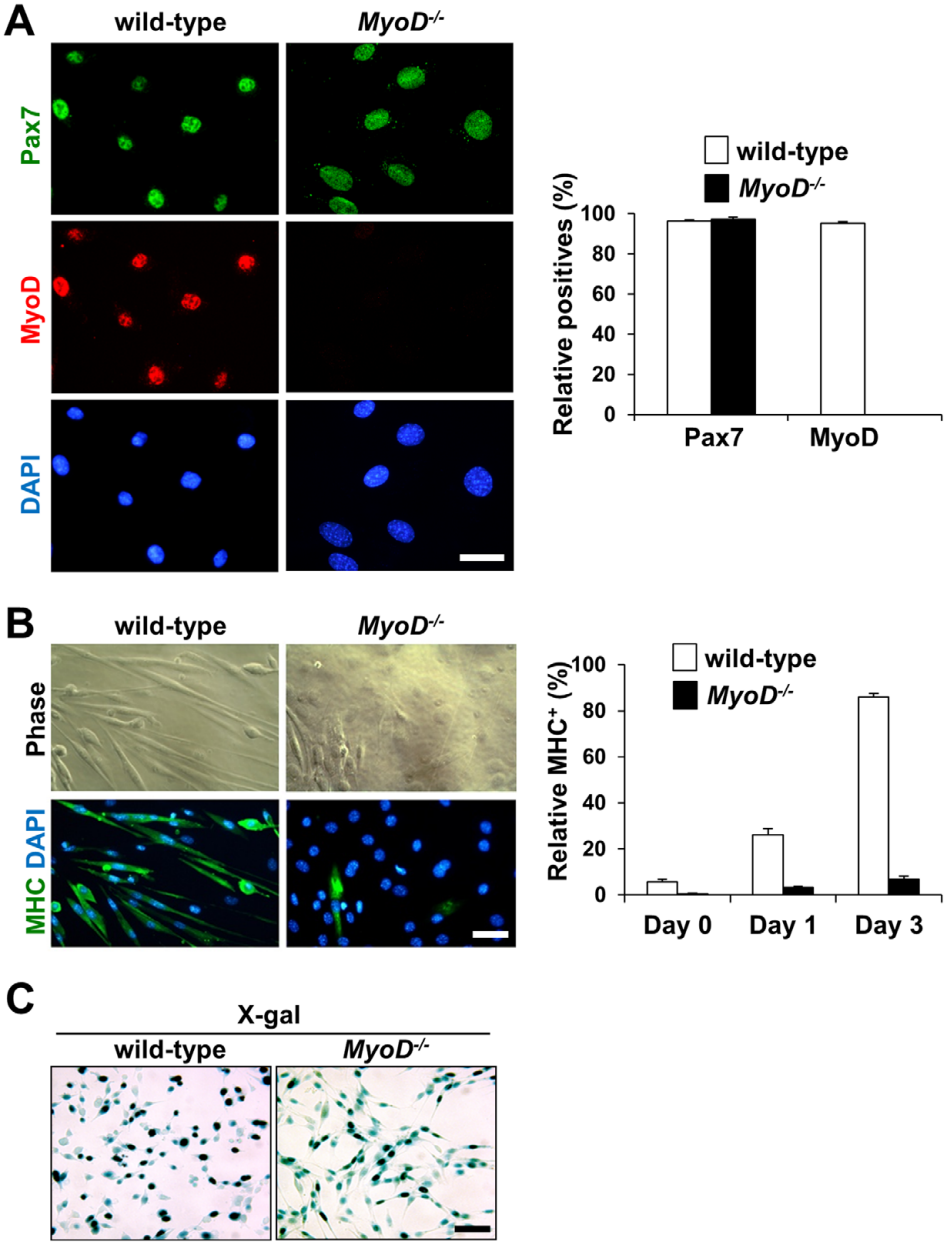
Expression of myogenic markers in wild-type and *MyoD^−/−^* myoblast cultures. (A) Fluorescence views of myoblasts isolated from wild-type and *MyoD^−/−^* mice. Immunostaining for Pax7 (green) and MyoD (red) showed that nearly all wild-type myoblasts expressed Pax7 and MyoD while *MyoD^−/−^* cells expressed only Pax7 (n = 3). Scale bar = 25 µm. (B) After 3 days of culture in low serum conditions, sarcomeric myosin heavy chain (MHC) immunostaining clearly demonstrated that wild-type myoblasts differentiated into MHC^+^ myocytes and myotubes while myogenic differentiation was delayed for *MyoD^−/−^* myoblasts (n = 3). Scale bar = 50 µm. (C) More than 90% of stable wild-type and *MyoD^−/−^* myoblast transformants expressed nuclear lacZ after co-transfection of PGK-nlacZ-MAR/PGK-puro followed by puromycin selection. Scale bar = 50 µm. Nuclei were counter-stained with DAPI (blue).

Recently, we reported that *MyoD^−/−^* myoblasts display remarkable resistance to apoptosis and increased cell survival compared to wild-type myoblasts after injection into injured skeletal muscle [29], [30]. To compare cell engraftment and cardiac function after the direct injection of myoblasts into infarcted mouse heart, wild-type and *MyoD^−/−^* myoblasts were purified from the skeletal muscle of adult mice and passaged 6–8 times before transplantation into infarcted hearts. *MyoD^−/−^* myoblasts displayed an enlarged cytoplasm and nuclear processes. In contrast, wild-type cells displayed a rounded morphology with a small, compact nucleus (Fig. 1A). Under growth conditions, *MyoD^−/−^* myoblasts clearly expressed the myogenic marker Pax7, but not the myogenic marker MyoD, while wild-type myoblasts expressed both markers (Fig. 1A). As previously reported, *MyoD^−/−^* myoblasts, but not wild-type myoblasts, displayed a severe defect in their ability to differentiate into sarcomeric myosin heavy chain (MHC)^+^ elongated myocytes or multinucleated myotubes under low serum conditions (Fig. 1B) [28]. However, *MyoD^−/−^* myoblasts were able to undergo terminal differentiation when incubation times in low serum conditions were increased [28] or cells were injected directly into injured muscle [29]. Therefore, we concluded that both wild-type and *MyoD^−/−^* myoblasts had been successfully isolated from adult mice. Following isolation, wild-type and *MyoD^−/−^* myoblasts were co-transfected with PGK-nlacZ-MAR and PGK-puro plasmids for cell transplantation, and stable transformants were pooled after puromycin selection. Fig. 1C shows that more than 90% of stable wild-type and *MyoD^−/−^* transformants expressed nuclear lacZ after puromycin selection.

### Efficient Engraftment of *MyoD^−/−^* Myoblasts after Transplantation into Infarcted Heart

**Figure 2:**
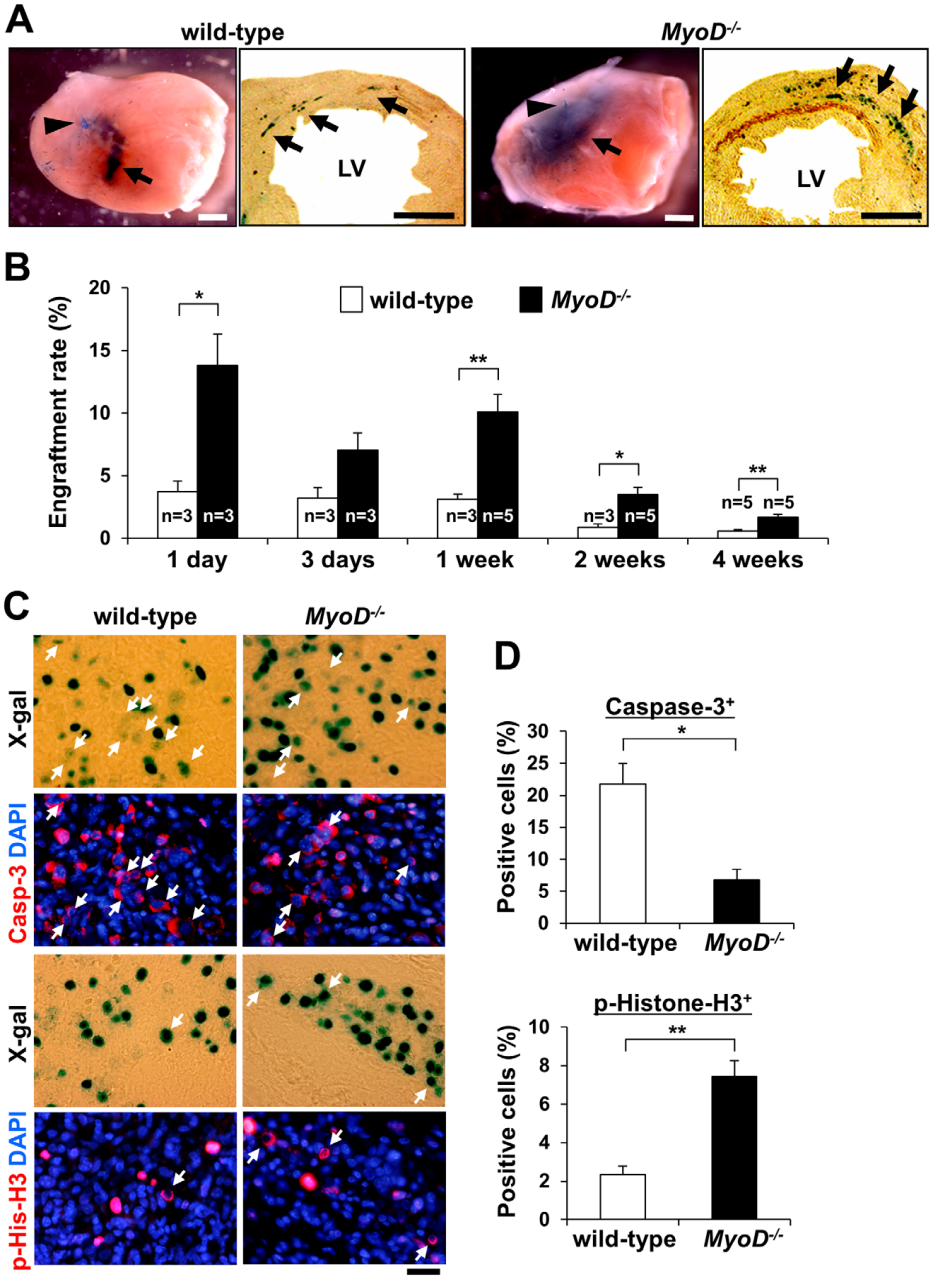
Engraftment of wild-type and *MyoD^−/−^* myoblasts after myocardial injection into infarcted heart. (A) These panels show MI induced by left coronary artery ligation. Wild-type and *MyoD^−/−^* myoblasts were directly injected into the peri-infarct regions of LV. After 1 week, X-gal staining of whole heart indicated that more *MyoD^−/−^* myoblasts engrafted than wild-type myoblasts (arrows). Arrowheads indicate left coronary artery ligation points. X-gal staining of cross sections indicated that more *MyoD^−/−^* myoblasts than wild-type myoblasts engrafted in both injured and uninjured areas of the heart. Arrows indicate engrafted lacZ^+^ wild-type and *MyoD^−/−^* myoblasts. Scale bars = 1 mm. (B) Engraftment of injected cells in the infarcted heart. More *MyoD^−/−^* myoblasts than wild-type myoblasts engraft in the infarcted heart 3 day to 4 weeks post-transplantation. Y-axis indicates the survival rates of engrafted cells after an injection of 1×10^6^ wild-type or *MyoD^−/−^* myoblasts. (C) By day 3, anti-activated caspase-3 (Casp-3) and anti-phospho-histone H3 (p-His-H3) antibody staining demonstrated that lacZ^+^ wild-type and *MyoD^−/−^* myoblasts proliferate or undergo apoptotic cell death (n = 4). Nuclei were counter-stained with DAPI (blue). Scale bar = 50 µm. (D) Comparison of the relative numbers of lacZ^+^/activated caspase-3^+^ or lacZ^+^/phospho-histone H3^+^ cells for wild-type and *MyoD^−/−^* myoblasts 3 days after transplantation.

Next, isolated myoblasts were used for transplantation after MI. One week after transplantation into the peri-infarct region, X-gal staining showed that *MyoD^−/−^* myoblasts engrafted at a higher rate in the recipient heart than wild-type myoblasts (Fig. 2A). Cross sections clearly showed that the progeny of wild-type and *MyoD^−/−^* myoblasts were visible at the site of injection, near the peri-infarct region of left ventricular (LV), and also in uninjured areas of the heart (Fig. 2A). Fig. 2B shows the myoblast engraftment rate by 1, 3, 7, 14 and 28 days after cell transplantation. As previously reported [32], both *MyoD^−/−^* and wild-type myoblast groups displayed a significant reduction in cell number 1 day after injection (*MyoD^−/−^* myoblasts 13.80±2.52%, n = 3, wild-type myoblasts 3.75±0.83%, n = 3, *p*<0.05). This reduction was more pronounced for wild-type myoblasts than for *MyoD^−/−^* myoblasts. Previous work demonstrated that *MyoD^−/−^* myoblasts possess remarkable resistance to apoptosis and increased survival compared to wild-type myoblasts after injection into regenerating skeletal muscle [29], [30]. Similar to myoblast transplantation into injured skeletal muscle, antibody staining for caspase-3 clearly showed massive apoptotic cell death of the engrafted myoblasts 3 days after transplantation (Fig. 2C, D). This cell death was more prominent for wild-type myoblasts than *MyoD^−/−^* myoblasts. Therefore, it is likely that the immediate reduction in cell number is due to post-transplantation myoblast cell death. In addition, engrafted *MyoD^−/−^* myoblasts displayed higher rates of proliferation by day 3 (Fig. 2C, D). After 1 week, significantly more *MyoD^−/−^* myoblasts engrafted than wild-type myoblasts (*MyoD^−/−^* myoblasts 10.08±1.41%, n = 5, wild-type myoblasts 3.11±0.43%, n = 3, *p*<0.05) (Fig. 2B). By weeks 2 and 4, the engraftment of *MyoD^−/−^* myoblasts declined but remained significantly higher than the engraftment of wild-type myoblasts (*MyoD^−/−^* myoblasts 3.51±0.59%, n = 5, wild-type myoblasts 0.87±0.29%, n = 3, *p*<0.05, and *MyoD^−/−^* myoblasts 1.68±0.23%, n = 5, wild-type myoblasts 0.59±0.12%, n = 5, *p*<0.01, respectively). These results clearly demonstrate that larger numbers of *MyoD^−/−^* myoblasts survive in the infarcted heart at least by 4 weeks post-transplantation, and that a decrease in cell death and increase in cell proliferation may contribute to their higher engraftment rates.

### Improved Cardiac Function after *MyoD^−/−^* Myoblast Transplantation

**Figure 3:**
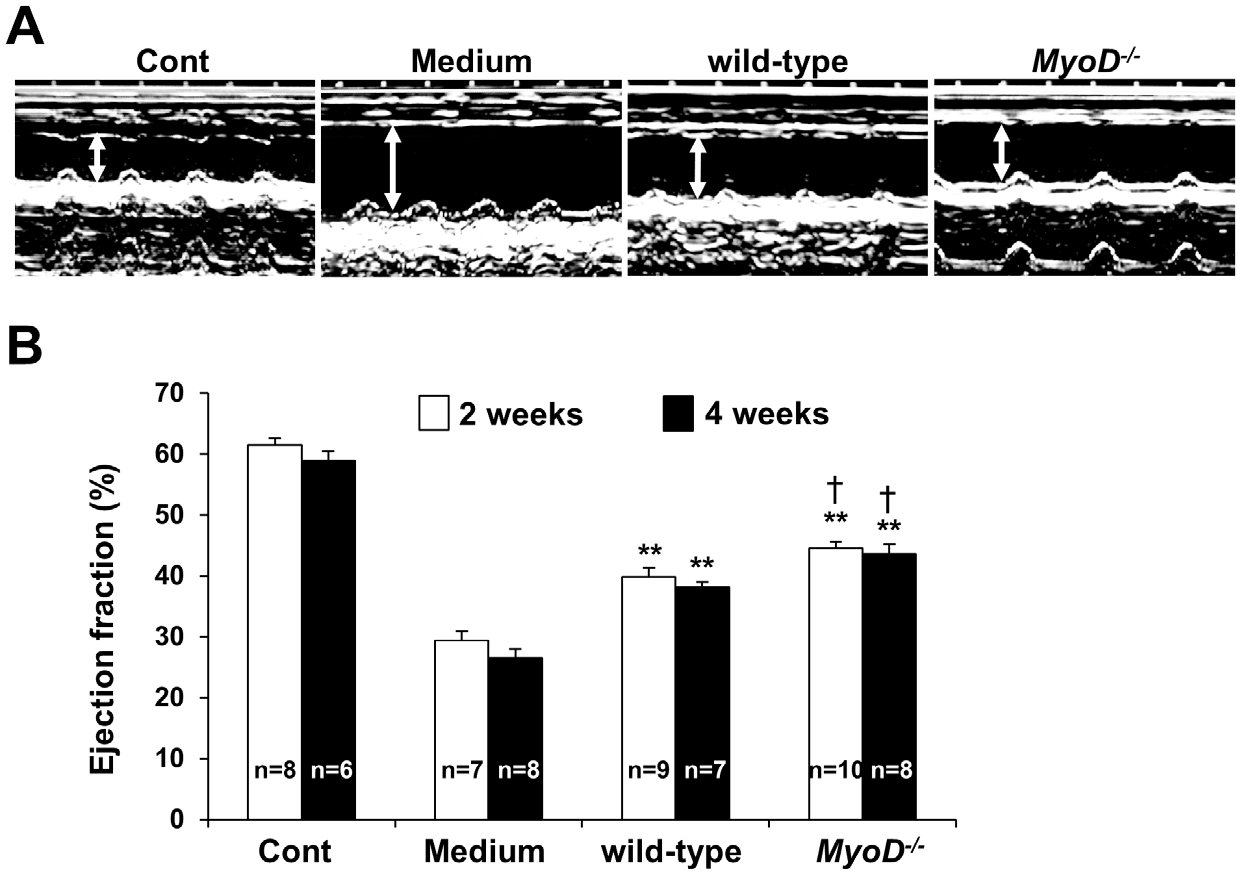
Improved cardiac function after myoblast transplantation. (A) Two weeks post-transplantation, M-mode echocardiograms were obtained for control mice and mice undergoing MI. Mice undergoing MI were transplanted with wild-type myoblasts, *MyoD^−/−^* myoblasts or medium alone. LV end-diastolic dimensions were significantly lower for mice receiving *MyoD^−/−^* and wild-type myoblasts compared to mice receiving medium alone. White arrows indicate LV end-diastolic dimensions. (B) Two and 4 weeks post-transplantation, echocardiograms demonstrated that ejection fractions for mice receiving wild-type or *MyoD^−/−^* myoblasts improved compared to mice receiving medium alone (double asterisks, *p*<0.01). Two weeks post-transplantation, mice receiving *MyoD^−/−^* myoblasts displayed an improved ejection fraction compared to mice receiving wild-type myoblasts (daggers, *p*<0.05). The control group did not undergo MI.

The higher engraftment rates of *MyoD^−/−^* myoblasts in recipient heart suggested that they may improve cardiac function more efficiently than wild-type myoblasts. Table 2 and Fig. 3A show the results of echocardiographic assessment 2 and 4 weeks after MI with or without cell transplantation. LV end-diastolic dimensions were significantly lower for hearts transplanted with *MyoD^−/−^* and wild-type myoblasts compared to hearts that received medium alone. In addition, LV end-systolic dimensions were also significantly lower at 2 and 4 weeks for mice receiving *MyoD^−/−^* and wild-type myoblasts compared to mice receiving medium alone. This result suggests that LV dilation seen in infarcted hearts receiving medium alone may have been improved as a result of myocardial myoblast transplantation. Myocardial infarction frequently produces LV dilation associated with myocyte hypertrophy and interstitial fibrosis outside the injured myocardium. These changes in LV geometry, referred to as remodeling, contribute to the development of depressed cardiac performance [33]. Importantly, the *MyoD^−/−^* group showed increased ejection fraction compared to the wild-type group by 2 and 4 weeks (*MyoD^−/−^* myoblasts 44.6±1.1%, n = 10, wild-type myoblasts 39.8±1.5%, n = 9, *p*<0.05, and *MyoD^−/−^* myoblasts 43.6±1.6%, n = 8, wild-type myoblasts 38.1±0.8%, n = 7, *p*<0.05, respectively) without any significant differences in the MI sizes among the 3 groups (Table 2 and Fig. 3B). These results suggest that *MyoD^−/−^* myoblasts improve cardiac systolic function more efficiently than wild-type myoblasts at least by 4 weeks after transplantation.

**Table 1:** Primer sequences used for RT-PCR.

Gene	Forward Primer	Reverse Primer	PCR Cycles	Product (bp)
MyoD	GGAGGAGCACGCACACTTC	CGCTGTAATCCATCATGCCATCAGAGC	25	464
CX3CL1	CAGTGGCTTTGCTCATCCGCTATC	ATGCTCTGAGGCTTAGCCGTAAGC	25	301
FGF7	GCTTCCACCTCGTCTGTCTAGTGG	CCCTCCGCTGTGTGTCCATTTAGC	25	431
BDNF	CAGCAGTCAAGTGCCTTTGGAGCC	GTTCGGCATTGCGAGTTCCAGTGC	25	342
VEGF-D	GAGGAGTTGCTGCAAATCGCGCAC	AACTCGGGCACTGATGTCAGAGGC	30	392
SCF	ACCCTCAACTATGTCGCCGGGATG	TCCTAAGGGAGCTGGCTGCAACAG	25	424
SDF-1	GTGTCCTCTTGCTGTCCAGCTCTG	AAGTTCCTCGGGCGTCTGACTCAC	35	365
PlGF	GCCTTTCAACGAAGTGTGGGGTCG	TCCTCTGAGTGGCTGGTTACCTCC	25	403
IGFBP2	CGGGTACCTGTGAAAAGAGACGCG	GGTTGTACCGGCCATGCTTGTCAC	30	411
IGFBP5	TGGGCTGTGAGCTGGTCAAAGAGC	CCCACAAACTTGGACTGGGTCAGC	25	373
HIF1α	CTTCTGGATGCCGGTGGTCTAGAC	CTCTCATTTCCTCATGGTCACATGG	30	223
β-actin	CACCCTGTGCTGCTCACCGAGGCC	ACCGCTCGTTGCCAATAGTGATGA	20	463

**Table 2:** Echocardiography Data and MI size

	Control	Medium	wild-type	*MyoD^-/-^*
**2 weeks (N)**	8	7	9	10
**Dd (mm)**	3.49 ± 0.05	4.54 ± 0.09	4.12 ± 0.08*	4.04 ± 0.10**
**Ds (mm)**	2.16 ± 0.04	3.81 ± 0.11	3.19 ± 0.08**	3.00 ± 0.06**
**EF (%)**	61.54 ± 1.07	29.39 ± 1.46	39.84 ± 1.51**	44.55 ± 1.09** †
**MI size (%)**	0	27.8 ± 4.3	27.5 ± 4.0	25.1 ± 4.2
**4 weeks (N)**	6	8	7	8
**LVDd (mm)**	3.64 ± 0.04	4.50 ± 0.03	4.22 ± 0.03**	4.06 ± 0.05**
**LVDs (mm)**	2.33 ± 0.07	3.86 ± 0.06	3.32 ± 0.04**	3.05 ± 0.07**
**EF (%)**	58.91 ± 1.53	26.48 ± 1.57	38.14± 0.81**	43.56 ± 1.59** †
**MI size (%)**	0	25.2 ± 3.9	24.5 ± 3.5	23.8 ± 4.9

LVDd; Left Ventricular End-Diastolic Dimension; LVDs; Left Ventricular End-Systolic Dimension; EF: Ejection Fraction, MI: Myocardial Infarction, *MyoD^-/-^*: *MyoD^-/-^* myoblasts, wild-type: wild-type myoblasts,

* p<0.05 vs. the medium group,

** p<0.01 vs. the medium group,

† p<0.05 vs. the wild-type myoblast group, Values are mean ± SEM.

### Skeletal Muscle Differentiation of Engrafted Myoblasts Following Transplantation

**Figure 4:**
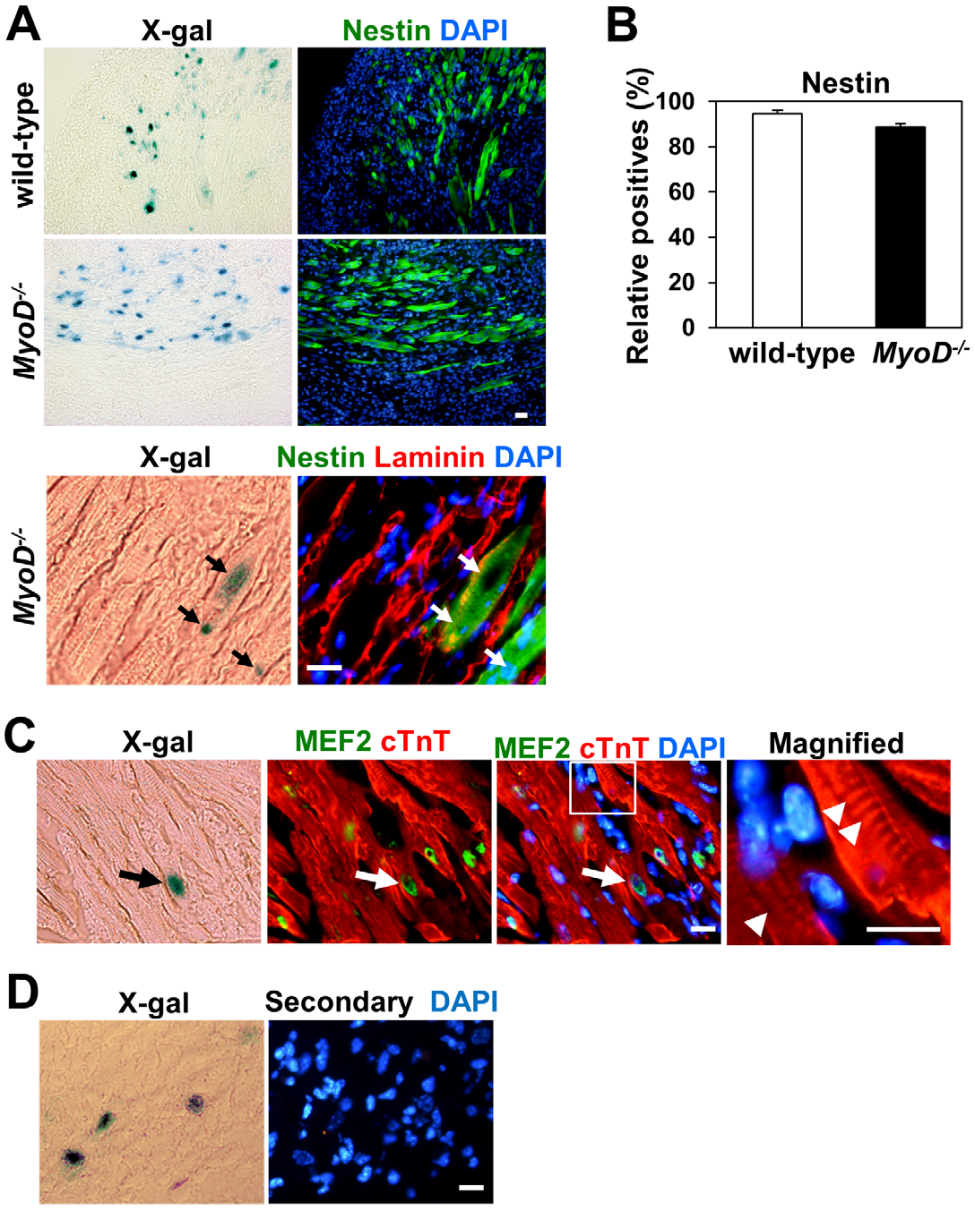
Skeletal muscle fiber differentiation of engrafted wild-type and *MyoD^−/−^* myoblasts following transplantation. (A) Two weeks post-transplantation, immunofluorescence staining of heart cross sections showed that the progeny of lacZ^+^ wild-type and *MyoD^−/−^* myoblasts formed nestin^+^ multinucleated skeletal myotubes. Laminin (red) indicates cardiomyocytes and skeletal myotubes. Arrows indicate lacZ^+^ donor cell-derived nuclei in nestin^+^ myotubes. (B) Comparison of the relative numbers of lacZ^+^/nestin^+^ myotubes for wild-type and *MyoD^−/−^* myoblasts 2 weeks after injection (n = 3). (C) Two weeks post-transplantation, immunofluorescence staining of heart cross sections showed lacZ^+^ *MyoD^−/−^* myoblast-derived cardiomyocyte-like cells express cardiac troponin T (cTnT, red) in their cytoplasm and myocyte enhancer factor 2 (MEF2, green) in their nucleus (arrows). (D) Secondary antibodies alone did not show any background staining. Nuclei were counter-stained with DAPI (blue). Scale bars = 50 µm.

Next, we examined the fate of engrafted myoblasts following myocardial injection (Fig. 4A, B). First, an anti-nestin antibody was used to detect skeletal muscle-derived cells after lacZ staining. Nestin is a component of intermediate filaments expressed in myoblasts and regenerating skeletal muscle fibers but not in the cardiomyocytes of adult mice [34], [35], [36]. The majority of lacZ-positive *MyoD^−/−^* and wild-type myoblast-derived progeny formed nestin-positive multinucleated skeletal myotubes in the infarcted heart by 2 weeks (wild-type myoblasts 94.7±1.6%, *MyoD^−/−^* myoblasts 88.7±1.5%, n = 3) (Fig. 4A, B). These nestin-positive skeletal myotubes also expressed laminin, a marker of the basal lamina in both skeletal muscle fibers and cardiomyocytes (Fig. 4A). To examine whether engrafted myoblasts can give rise to cardiomyocytes in recipient hearts, we performed immunostaining against several cardiomyocyte markers 2 weeks after cell transplantation. Sections were examined to identify nuclear lacZ/DAPI positive cells co-expressing cardiomyocyte markers. Very few cells derived from *MyoD^−/−^* myoblasts co-expressed the cardiac sarcomeric protein cardiac-troponin T (cTnT) and the cardiac transcription factor myocyte enhancer factor-2 (MEF2) after transplantation (Fig. 4C). These results suggest that the majority of engrafted wild-type and *MyoD^−/−^* myoblasts form skeletal muscle fibers upon transplantation into damaged heart tissue. Therefore, improved cardiac function in infarcted heart following myoblast transplantation is not due to the contribution of cardiomyocyte-like cells.

### Increased Angiogenesis after Myoblast Transplantation in Infarcted Heart

**Figure 5:**
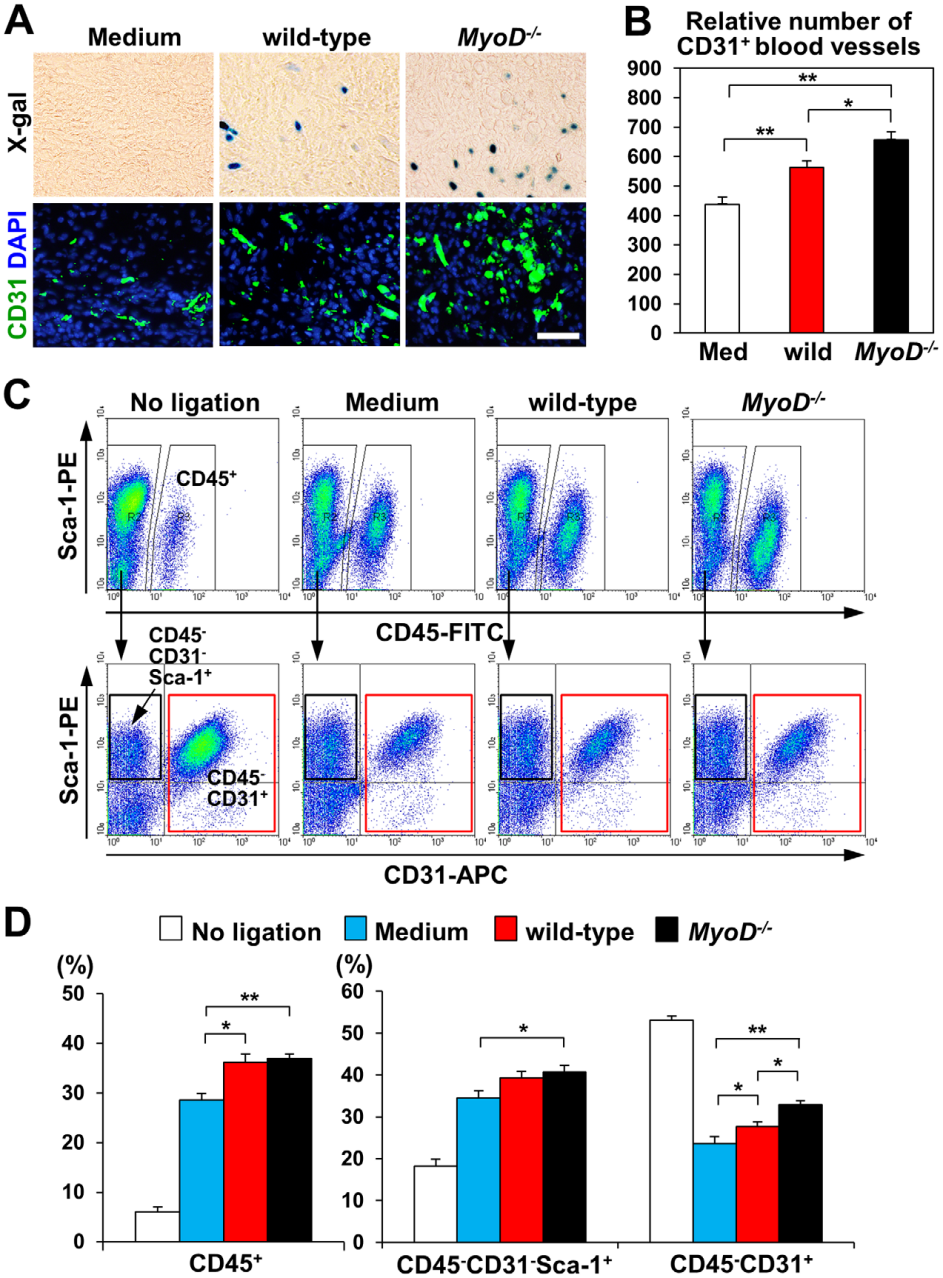
Increased angiogenesis in scar area after myocardial injection of myoblasts. (A) Representative images of heart sections 1 week after cell injection. X-gal positive cells in the scar area indicate the progeny of injected cells. Green fluorescence indicates CD31^+^ endothelial cells in the vasculature. Nuclei were counter-stained with DAPI (blue). Scale bar = 50 µm. (B) Vascular density was measured using anti-CD31 antibody staining of scar tissue cross sections 1 week after injection of wild-type myoblasts, *MyoD^−/−^* myoblasts, or medium (n = 3). (C) FACS analysis showed an increase in the number of CD45^+^ blood cells (arrows) and CD45^ -^ CD31^−^ Sca-1^+^ cardiac progenitor cells (black boxes) and a decrease in the number of CD45^−^ CD31^+^ endothelial cells (red boxes) in the hearts of mice receiving wild-type myoblasts, *MyoD^−/−^* myoblasts, or medium alone. (D) The total number of CD45^+^ blood cells, CD45^ -^ CD31^−^ Sca-1^+^ cardiac progenitors, and CD45^−^ CD31^+^ endothelial cells was estimated using FACS data (n = 3).

Recent work suggests that a variety of stem cell types can repair heart function through paracrine effects that induce angiogenesis in the injured heart [22], [3]. Therefore, we examined whether the injection of myoblasts could increase angiogenesis 1 week after transplantation (Fig. 5A, B). The number of CD31^+^ blood vessels in the injured area was greater after transplantation of *MyoD^−/−^* myoblasts (656.8±27.0%, n = 8) than after transplantation of wild-type myoblasts (562.8±23.2%, n = 8) or medium alone (437.3±25.1%, n = 7). FACS analysis was performed to measure the number of CD45^−^ CD31^+^ endothelial cells 1 week after MI (Fig. 5C, D). Clearly, MI injected with medium alone induced a marked decrease in the number of CD45^−^ CD31^+^ endothelial cells compared to sham heart (no ligation) (Medium: 22.7±1.7%, No ligation, 53.1±2.7%, n = 6). Myoblast injection increased the number of CD45^−^ CD31^+^ endothelial cells in the infarct and border zones. In addition, the number of CD45^−^ CD31^+^ endothelial cells in the infarct and border zones was greater for the *MyoD^−/−^* myoblast group than for the wild-type myoblast group (*MyoD^−/−^* myoblasts 32.9±1.0%, wild-type myoblasts 27.7±1.0%, n = 6, *p*<0.05). The infiltration of blood cells and cardiac progenitor cells is known to improve heart histology and function after MI [3]. Therefore, we used FACS analysis to measure the infiltration of CD45^+^ blood cells and CD45^−^ CD31^−^ Sca-1^+^ cardiac progenitor cells (Fig. 5C, D). Following MI, an increase in the infiltration of CD45^+^ and CD45^−^ CD31^−^ Sca-1^+^ cells was detected. However, the number of CD45^+^ and CD45^−^ CD31^−^ Sca-1^+^ cells was not significantly different between wild-type and *MyoD^−/−^* myoblast groups.

### Co-culture with *MyoD^−/−^* Myoblasts Stimulates Cell Proliferation of Endothelial Cells

**Figure 6:**
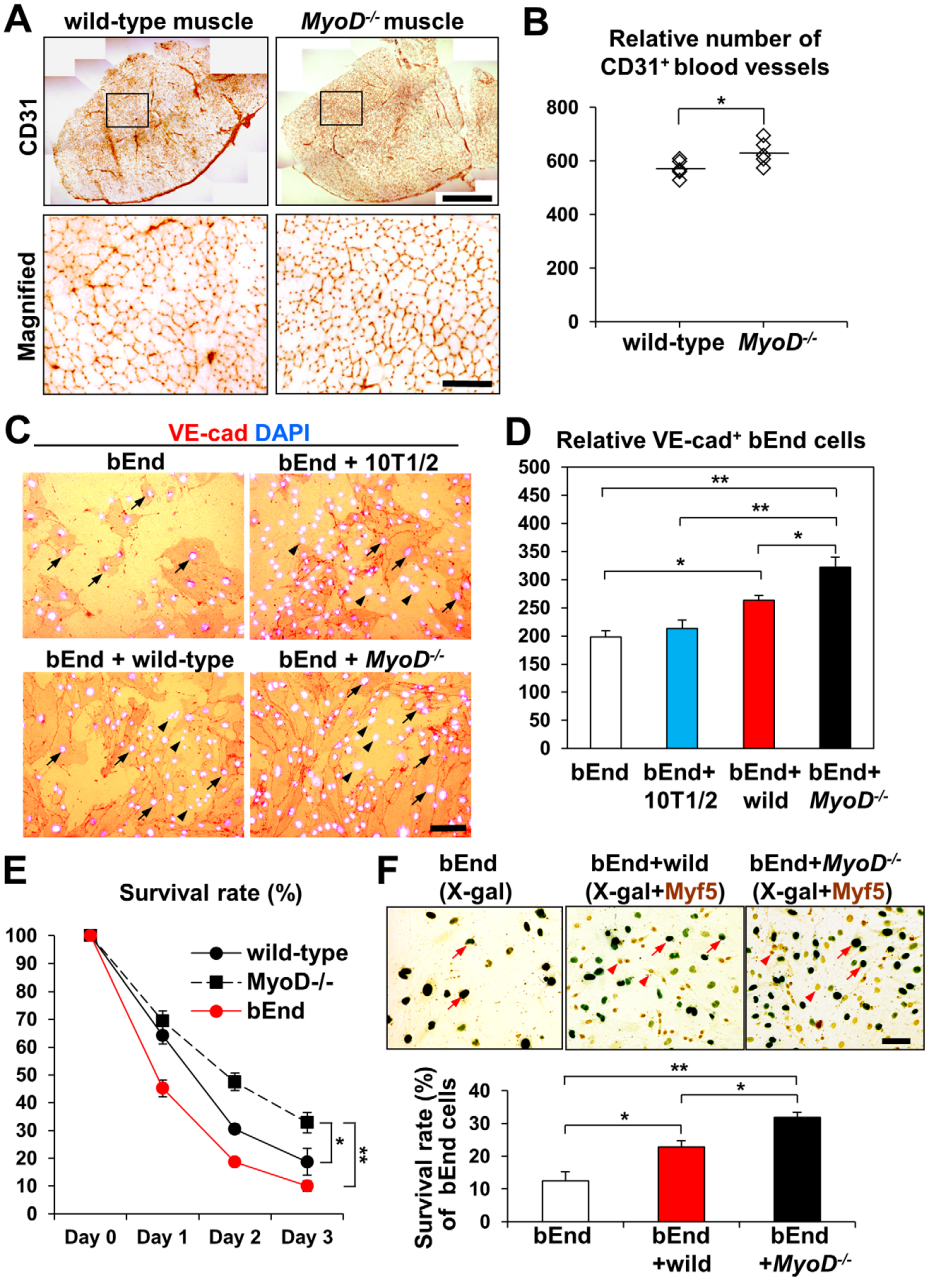
Co-culture with *MyoD^−/−^* myoblasts stimulates cell proliferation of endothelial cells. (A) Upper panels: Whole tibialis anterior muscle cross sections of wild-type and *MyoD^−/−^* adult skeletal muscle after anti-CD31 antibody staining. Scale bar = 500 µm. Lower panels: Magnified views from upper sections. Rectangles in upper panels indicate the magnified view areas. Scale bar = 100 µm. (B) Vascular density was measured using anti-CD31 antibody staining of cross sections (n = 5). Bars indicate averages for each group. (C) VE-cadherin (VE-cad) staining of bEnd cells (arrows) after 3 days of co-culture with 10T1/2 fibroblasts cells (arrowheads), wild-type myoblasts (arrowheads) or *MyoD^−/−^* myoblasts (arrowheads). Scale bar = 50 µm. Nuclei were counter-stained with DAPI (white). (D) Quantification of bEnd cell proliferation after 3 days of co-culture measured as the number of VE-cadherin (VE-cad) positive cells in culture (n = 3). (E) bEnd cells, wild-type and *MyoD^−/−^* myoblasts were cultured under normal and hypoxic conditions for 1–3 days (n = 3). (F) bEnd cells (red arrows) were co-cultured for 3 days with Myf5-expressing wild-type or *MyoD^−/−^* myoblasts (red arrowheads) under hypoxic conditions. Percentile indicates the relative number of X-gal positive bEnd cells per seeded cell (n = 4). Scale bar = 50 µm.

*MyoD^−/−^* myoblast transplantation induced greater angiogenesis in infarcted heart compared to wild-type myoblasts, suggesting that the *MyoD^−/−^* muscle niche may promote greater endothelial cell proliferation and/or survival compared to wild-type muscle. To examine this possibility, we compared vascular densities between wild-type and *MyoD^−/−^* adult tibialis anterior skeletal muscles. The *MyoD^−/−^* muscle niche clearly displayed a larger number of CD31^+^ blood vessels compared to the wild-type muscle niche (*MyoD^−/−^*631.6±21.1%, wild-type 572.6±14.2%, n = 5, *p*<0.05) (Fig. 6A, B). To test for possible interactions between this skeletal muscle niche and endothelial cells, brain-derived endothelial (bEnd) cells were either cultured alone or co-cultured with mouse 10T1/2 fibroblasts, wild-type myoblasts or *MyoD^−/−^* myoblasts (Fig. 6C, D). Cells were grown in myoblast growth medium for 3 days and stained for the endothelial cell marker VE-cadherin. bEnd cells co-cultured with *MyoD^−/−^* myoblasts (322.3±11.2%, n = 3) displayed more VE-cadherin positive endothelial cells than bEnd cells co-cultured with wild-type myoblasts (263.3±9.5%, n = 3) or 10T1/2 cells (213.0±15.5%, n = 3), indicating that co-culture with *MyoD^−/−^* myoblasts promotes endothelial cell proliferation. Taken together, these results strongly suggest that transplantation of *MyoD^−/−^* myoblasts promotes endothelial cell proliferation and increases angiogenesis via the secretion of growth factors by *MyoD^−/−^* myoblasts.

### *MyoD^−/−^* Myoblasts and Endothelial Cells Co-cultured with *MyoD^−/−^* Myoblasts Display Increased Cell Survival Under Hypoxic Conditions

To examine whether *MyoD^−/−^* myoblasts are resistant to hypoxia, and whether myoblasts are able to protect endothelial cells from cell death in an ischemic environment, wild-type and *MyoD^−/−^* myoblasts were co-cultured with bEnd cells incubated under hypoxic conditions for 3 days (Fig. 6E, F). While cell numbers for both wild-type and *MyoD^−/−^* myoblasts decreased over time under hypoxic conditions, survival of *MyoD^−/−^* myoblasts was substantially higher (32.8±3.7%, n = 3 by day 3) compared to wild-type myoblasts (18.7±4.8%, n = 3, *p*<0.05 by day 3) (Fig. 6E). Therefore, *MyoD^−/−^* myoblasts are more resistant to hypoxia than wild-type myoblasts, which may account for the higher survival rate of *MyoD^−/−^* myoblasts after injection into ischemic heart. In contrast, bEnd cells are more sensitive to hypoxic culture conditions by day 3 (10.0±1.9%, n = 3) (Fig. 6E). Interestingly, bEnd cells had a significantly higher survival rate when co-cultured with *MyoD^−/−^* myoblasts (31.9±1.6%, n = 4, *p*<0.01) and wild-type myoblasts (22.9±1.8%, n = 4, *p*<0.05) compared with bEnd cells alone (12.5±2.8%, n = 4). Co-culture with *MyoD^−/−^* myoblasts led to increased rates of bEnd survival compared to wild-type myoblast co-culture (*p*<0.05, Fig. 6F). These results suggest that transplantation of *MyoD^−/−^* myoblasts can efficiently improve cardiac systolic function by increasing angiogenesis, as well as endothelial cell proliferation and survival.

### Paracrine Angiogenic Factors Expressed in *MyoD^−/−^* Myoblasts

**Figure 7:**
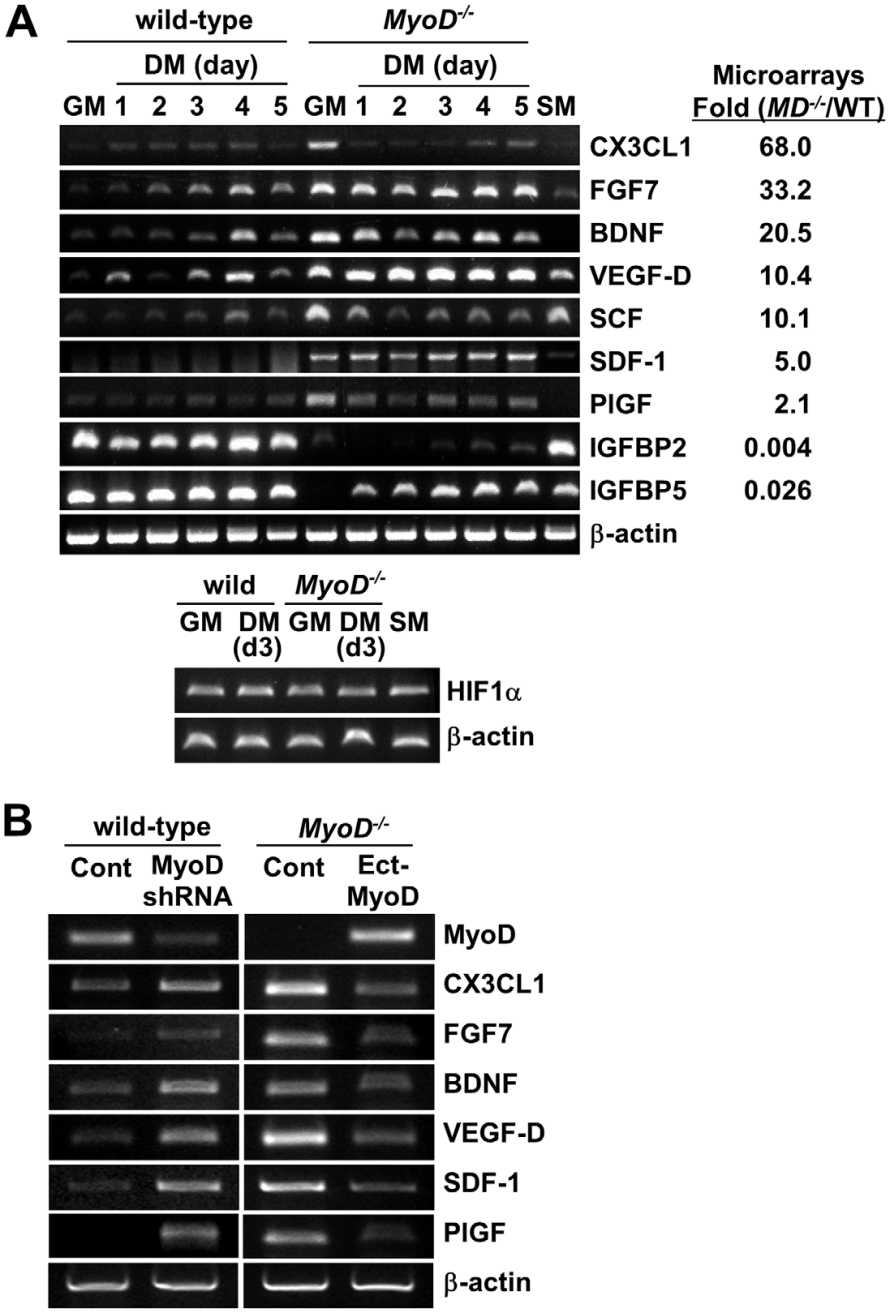
Angiogenic factors expressed in *MyoD^−/−^* myoblasts. (A) Expression of growth factor related genes in wild-type and *MyoD^−/−^* myoblasts was confirmed by semi-quantitative RT-PCR. RNA was isolated from wild-type and *MyoD^−/−^* myoblasts grown in growth medium (GM), differentiation medium (DM), or under skeletal muscle (SM) culture conditions for 1–5 days. Microarray data from Table 2 was shown at the right side. The expression of HIF1α was also examined for wild-type and *MyoD^−/−^* myoblasts. (B) RT-PCR comparison of the expression of angiogenic genes between wild-type myoblasts infected with a lentiviral vector expressing shRNA for MyoD-KD and a control shRNA vector (Cont), or between *MyoD^−/−^* myoblasts infected with a lentiviral vector expressing MyoD (Ect-MyoD) and an empty control vector (Cont). β-actin was used as a loading control.

To identify which molecular cascades are altered in *MyoD^−/−^* myoblasts, we re-visited data generated from Affymetrix GeneChip DNA microarrays [29]. Previously, we demonstrated that anti-apoptotic genes are up-regulated in *MyoD^−/−^* myoblasts, while genes known to execute apoptosis are down-regulated [29]. Table 3 and Fig. 7A show that *MyoD^−/−^* myoblasts up-regulate a number of growth factors including the angiogenic factors chemokine CXC3 motif ligand 1 (CX3CL1), fibroblast growth factor 7 (FGF7), brain-derived neurotrophic factor (BDNF), vascular endothelial growth factor-D (VEGF-D), stem cell factor (SCF), stromal cell-derived factor-1 (SDF-1) and placental growth factor (PlGF) but down-regulate other growth factors such as insulin like growth factor binding protein 2 (IGFBP2) and 5 (IGFBP5). CX3CL1 is a chemokine involved in inflammation [37]. FGF7 is a mitogen for epithelial cells involved in wound healing [38]. BDNF is a trophic factor for neuronal development and synaptic plasticity [39]. SCF is a cyctokine for hematopoiesis [40]. VEGF-D and PlGF are a VEGF family involved in vasculogenesis and angiogenesis [41]. SDF-1 is involved in the chemo-attraction of many types of stem cells [42]. IGFBP2 and 5 are binding partners of insulin like growth factor (IGF), which inhibits IGF activity [43]. We confirmed the GeneChip microarray data for these growth factor-related genes using semi-quantitative RT-PCR (Fig. 7A). These results suggested that *MyoD^−/−^* myoblasts have a greater survival rate after transplantation due to alterations in apoptotic cascades, the expression of angiogenic factors, and the induction of angiogenesis. We also examined HIF1α expression, a hypoxia inducible transcription factor that regulates VEGF gene expression. However, there was no difference in HIF1α gene expression between wild-type and *MyoD^−/−^* myoblasts.

**Table 3:** Different expression in secreted factor genes in between *MyoD^-/-^* and wild-type myoblasts.

GenBank	UniGene	Symbol		Gene Name (Secreted Molecule)	Fold*
**U92565**	**Mm.103711**	**Cx3cl1**	**Chemokine (CX3C motif) ligand 1 (CL3CL1)** †		**68.0**
**Z22703**	**Mm.330557**	**Fgf7**	**Fibroblast growth factor 7 (FGF7)** †		**33.2**
M70642	Mm.1810	Ctgf	Connective tissue growth factor, IGFBP8		32.3
**X55573**	**Mm.1442**	**Bdnf**	**Brain derived neurotrophic factor (BDNF)** †		**20.5**
**X99572**	**Mm.297978**	**Figf**	**Fos-induced growth factor, vascular endothelial growth factor D (VEGF-D)** †		**10.4**
**U44725**	**Mm.45124**	**Kitl**	**Kit ligand, Stem cell factor (SCF)** †		**10.1**
L02914	Mm.18625	Aqp1	Aquaporin 1		8.0
L75822	Mm.233470	Igfbp7	Insulin-like growth factor binding protein 7		7.0
**L12030**	**Mm.303231**	**Cxcl12**	**Chemokine (CXC motif) ligand 12, Stromal cell derived factor 1 (SDF-1)** †		**5.0**
W89821	Mm.42249	Neo1	Neogenin		3.6
X81584	Mm.358609	Igfbp6	Insulin-like growth factor binding protein 6		2.9
D63663	Mm.7411	Hdgfl1	Hepatoma derived growth factor-like 1		2.8
X85990	Mm.4083	Sema3b	short basic domain, secreted, Semaphorin 3B		2.8
X85993	Mm.372039	Sema3a	short basic domain, secreted, Semaphorin 3A		2.6
M86736	Mm.1568	Grn	Granulin		2.5
U55060	Mm.341434	Lgals9	Lectin, galactose binding, soluble 9		2.5
C75973	Mm.236969	Renbp	Renin binding protein		2.3
**X80171**	**Mm.4809**	**Pgf**	**Placental growth factor (PlGF)** †		**2.1**
AA120463	Mm.287977	Ecgf1	Platelet-derived endothelial cell growth factor 1 (PD-ECGF)		2.0
AA266467	Mm.275909	Sema4b	transmembrane/short cytoplasmic domain, Semaphorin 4B		2.0
AA408818	Mm.134093	Sema3e	short basic domain, secreted, Semaphorin 3E		0.333
X73580	Mm.4723	Sct	Secretin		0.043
**L12447**	**Mm.309617**	**Igfbp5**	**Insulin-like growth factor binding protein 5 (IGFP5)** †		**0.026**
**L05439**	**Mm.141936**	**Igfbp2**	**Insulin-like growth factor binding protein 2 (IGFP2)** †		**0.004**

* Mean fold change for pair wise comparisons of *MyoD^-/-^*/ wild-type / myoblasts.

† Genes highlighted by bold letters were chosen for further analysis shown in Figure 7A.

Next, we examined whether the acute loss of MyoD in wild-type myoblasts by gene knockdown (KD) is sufficient to induce expression of these angiogenic factors and whether ectopic expression of MyoD in *MyoD^−/−^* myoblasts can suppress expression of these genes. RT-PCR showed that wild-type myoblasts infected with a lentiviral vector expressing shRNA for MyoD exhibited an extensive reduction in MyoD expression compared to myoblasts infected with a control shRNA vector (Fig. 7B). Importantly, for wild-type myoblasts expression of angiogenic genes (CX3CL1, FGF7, BDNF, VEGF-D, SDF-1 and PlGF) was efficiently induced by MyoD-KD. By contrast, RT-PCR showed that infection with a lentiviral vector expressing MyoD effectively rescued MyoD expression in *MyoD^−/−^* myoblasts (Fig. 7 B). Ectopic expression of MyoD in *MyoD^−/−^* myoblasts resulted in a marked reduction in the expression of these angiogenic factors. These results suggest that MyoD is a negative regulator of these angiogenic genes.

### SDF-1 and PlGF can Stimulate Endothelial Cell Proliferation and Survival

**Figure 8:**
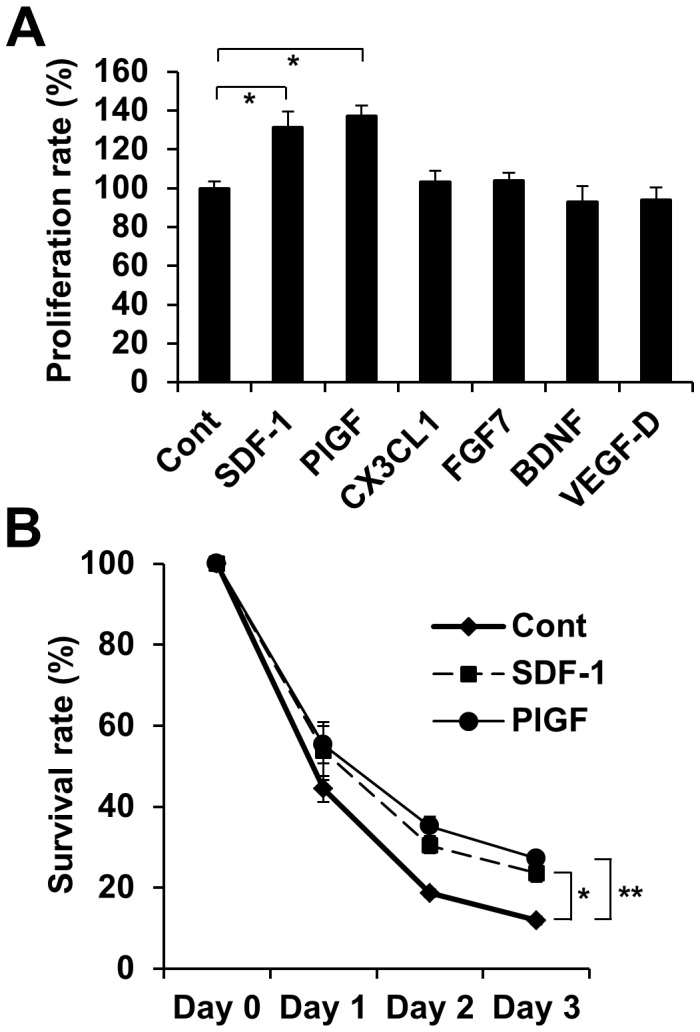
Angiogenic factors up-regulated in *MyoD^−/−^* myoblasts for endothelial cell proliferation and survival. (A) bEnd cells were cultured in the presence or absence of angiogenic growth factors for 3 days and the number of VE-cadherin positive endothelial cells was determined (n = 3). (B) bEnd cells were cultured in the presence or absences of SDF-1 or PlGF under hypoxic conditions for 1–3 days. The percentile indicates the relative number of bEnd cells for each time course (n = 3).

Microarray data and RT-PCR demonstrated that CX3CL1, FGF7, BDNF, VEGF-D, SDF-1 and PlGF genes are up-regulated in *MyoD^−/−^* myoblasts. To examine whether these angiogenic factors are able to stimulate angiogenesis, bEnd cells were cultured with these angiogenic factors for 3 days (Fig. 8A). Incubation with SDF-1 and PlGF had a stronger angiogenic effect (131.5±8.0% n = 3, *p*<0.05 and 137.3±5.4% n = 3, *p*<0.05, respectively) compared to control (100±3.6% n = 3). Next, to test whether SDF-1 and PlGF have an influence on the cell survival of cultured endothelial cells, bEnd cells were cultured in medium containing the angiogenic factors under hypoxic conditions for 1–3 days (Fig. 8B). Under hypoxic conditions, SDF-1 and PlGF induced greater survival of bEnd cells (by day 3, 23.6±2.1% n = 3, *p*<0.05 and 27.3±1.3% n = 3, *p*<0.01, respectively) compared to culture medium alone (by day 3, 11.9±1.0% n = 3). These results suggest that *MyoD^−/−^* myoblasts are able to improve cardiac function after transplantation into infarcted heart due, in part, to the secretion of the paracrine angiogenic factors SDF-1 and PlGF and the resulting induction of angiogenesis in the area of injury.

## Discussion

Patient specific skeletal myoblasts can be isolated using muscle biopsies and expanded *ex vivo* before autologous cell transplantation. In addition, myoblasts have been shown to engraft and form skeletal muscle fibers in recipient hearts. Although myoblasts are unable to couple electromechanically with the host myocardium, they can partially improve heart function by increasing angiogenesis, decreasing fibrosis, and directing the differentiation of other muscle fibers [44], [45], [12].

MyoD, a skeletal muscle-specific master transcription factor, plays an essential role in myogenic determination, and myoblasts lacking MyoD may represent an intermediate stage between quiescent satellite cells and myogenic precursors. Recently, our laboratory demonstrated that *MyoD^−/−^* myoblasts possess remarkable resistance to apoptosis and increased cell survival compared to wild-type myoblasts after injection into injured skeletal muscle [28], [29], [30].

In this study, we demonstrate that *MyoD^−/−^* myoblasts are a useful cell source for myocardial repair, and that they display higher rates of survival than wild-type myoblasts *in vivo* following transplantation into an infarcted heart, as well as *in vitro* under hypoxic conditions. Recently, Di Carlo et al. showed that hypoxia-dependent inhibition of skeletal muscle differentiation was associated with MyoD degradation by the ubiquitin-proteasome pathway [46]. In addition, hypoxic conditions inhibit permanent withdrawal of myoblasts from the cell cycle by producing a marked decrease in MyoD, Myf5 and myogenin levels. Recently, we demonstrated that *MyoD^−/−^* myoblasts up-regulate many anti-apoptotic genes including Pax3, Bcl-2 and Bcl-xL. It is possible that the delayed differentiation exhibited by *MyoD^−/−^* myoblasts protects against the high levels of apoptotic cell death displayed by wild-type myoblasts undergoing terminal differentiation [29], [30], [47]. In addition to an increase in cell survival, we have shown that transplantation of *MyoD^−/−^* myoblasts provides functional benefits to damaged heart tissue. Mice receiving *MyoD^−/−^* myoblasts displayed a greater improvement in ejection fraction by 4 weeks after transplantation, compared to the wild-type myoblasts. In the future, it will be important to determine how long this functional benefit lasts.

Transplantation of skeletal myoblasts into infarcted heart has consistently been shown to improve cardiac function [12]. Transplanted myoblasts survive, proliferate, and eventually differentiate into skeletal muscle fibers but fail to produce cardiomyocytes. In accordance with previous studies, we report here that engrafted myoblast progeny lack the expression of cardiac specific markers, and the majority of *MyoD^−/−^* myoblast-derived cells express nestin and laminin, markers of skeletal muscle myotube differentiation. Although recent work demonstrated that Wnt11 gene transfer enhances cardiomyogenic differentiation of skeletal muscle-derived stem cells [48], it is likely that engrafted myoblasts express cardiomyocyte-markers due to fusion with host cardiomyocytes in the recipient heart [49].

Although mechanisms leading to functional heart improvement remain unclear, transplanted myoblasts may initiate remodeling of the host tissue by exerting important paracrine effects on the surrounding myocardium [50], [51], [45], [23], [44]. It is possible that transplanted myoblasts, which eventually differentiate into multinucleated skeletal muscle fibers, also exert active contractile effects [52]. However, since skeletal muscle differentiation initiated after myocardial injection may induce cardiac arrhythmia [53], it needs to be cautious to use of skeletal myoblasts for myocardial injection. Importantly, the paracrine effects of angiogenic factors, rather than cardiomyocyte differentiation, must play an important role in cell-based therapy for infarcted heart [54], [55]. Cells shown to produce this result upon transplantation include skeletal myoblasts [44], [45], [56], [23], bone marrow-derived cells [3], [57], [58], mesenchymal stem cells [59], [60], human CD133^+^ cells [61], human cord blood progenitors [62] and human CD34^+^ cells [63]. The release of paracrine factors by transplanted cells may exert a protective effect by stimulating angiogenesis within the infarcted and non-infarcted regions. In turn, this increased angiogenesis may prevent cardiomyocytes from undergoing apoptotic cell death, protecting the infarcted heart from further cardiac remodeling and scar tissue formation.

Paracrine factors provide protection to injured heart by eliciting VEGF production, increasing vascular density, increasing blood flow, and decreasing endothelial cell apoptosis [64], [65]. PlGF, a growth factor belonging to the VEGF family, plays a critical role in pathologic angiogenesis. Recent work demonstrated that PlGF expression is up-regulated after myocardial damage, and that PlGF administration can facilitate cardiac repair after myocardial ischemia [66]. In addition, SDF-1 has been shown to induce stem cell homing to injured heart post-MI and induce angiogenesis [67], [68]. An interaction between satellite cells and endothelial cells within skeletal muscle was recently reported [69], and it is possible that reciprocal signals from satellite and endothelial cells may be required for appropriate *in vivo* cellular function.

Importantly, our work demonstrates that hearts receiving *MyoD^−/−^* myoblasts display an increase in vascular density compared to hearts receiving wild-type myoblasts or medium alone. These results suggest that transplantation of *MyoD^−/−^* myoblasts can efficiently improve cardiac systolic function through increased angiogenesis. Supporting this idea, Affymetrix DNA microarrays and RT-PCR demonstrated that *MyoD^−/−^* myoblasts up-regulate a number of growth factors including the angiogenic factors CX3CL1, FGF7, VEGF-D, SDF-1 and PlGF. In addition, *in vitro* cell culture experiments showed that SDF-1 and PlGF increase endothelial cell survival and proliferation. MyoD directly regulates the transcription of microRNA expression, suppressing specific targets during myogenic differentiation by blocking protein translation and/or degrading RNA, and it is possible that angiogenic genes up-regulated in *MyoD^−/−^* myoblasts contain MyoD-regulated microRNA binding sites [70], [30]. Regardless, the substantial increase in the survival of *MyoD^−/−^* myoblasts after transplantation provides reason to believe they may improve cardiac function more efficiently than their wild-type counterparts.

Recently, Menasche’s group demonstrated that low myoblast doses did not improve the regional or global LV function of patients with MI occurring 4 weeks before transplantation [71]. Notably, high doses of myoblasts significantly reduced LV volumes compared to the placebo group, indicating the importance of protecting LV remodeling after myoblast transplantation. However, we used an acute injury model for myoblast transplantation, which differs significantly from Menasche’s clinical trials.

Although the generation of human *MyoD^−/−^* myoblasts for clinical purposes is not possible, MyoD expression or function may be chemically, transcriptionally or post-translationally suppressed in order to recreate the beneficial effects of *MyoD^−/−^* myoblast transplantation shown here. Bromodeoxyuridine (BrdU), a thymidine analogue, is known to be a strong inhibitor of muscle differentiation through its suppression of *MyoD* gene expression [72]. Alternatively, suppression of MyoD may be achieved by infection of patient myoblasts with a lentivirus carrying an RNA interference (RNAi) or Id protein expression vector [30]. Id proteins are essential dimerization partners of the MyoD family of transcription factors, and act to suppress their effector functions [73].

In conclusion, our results suggest that transplanted *MyoD^−/−^* myoblasts are superior to wild-type myoblasts for myocardial cell therapy. These results represent a novel approach to myoblast transplantation, in which the ablation of MyoD expression increases angiogenesis, cell survival, and the functional recovery of infarcted heart.

## Materials and Methods

### Animals

Eight to 12-week-old BALB/c female mice weighing 17–20 g were obtained from Harlan Sprague Dawley. BALB/c wild-type and *MyoD^−/−^* myoblasts were transplanted into BALB/c recipient mice since immune rejection in this system has been reported to be minimal in short term transplantation experiments [74]. The animals were housed in an SPF environment and were monitored by the Research Animal Resources (RAR) of the University of Minnesota. All protocols were approved by the Animal Care and Use Committee (IACUC, Code Number: 1003A79635) of the University of Minnesota.

### Myoblast Isolation and Cell Culture

Isolation of satellite cell-derived primary myoblasts was performed as previously described [28], [75], [76]. Briefly, hind limb muscles from 1 to 2-month-old wild-type or *MyoD^−/−^* mice were digested with collagenase type B and dispase II (Roche Diagnostics). Primary myoblasts were cultured in growth medium consisting of HAM’s F-10 supplemented with 20% fetal bovine serum (FBS) and 5 ng/mL basic fibroblast growth factor (bFGF, Invitrogen) on collagen-coated dishes, as previously described [75], [76], [28]. After 4–6 passages, wild-type and *MyoD* ^−/−^ myoblasts were transfected with Lipofectamine-2000 (Invitrogen) and a 1∶10 ratio of PGK-nlacZ-MAR and PGK-puro plasmids. The stable transformants were pooled after 10 days of selection in 2 µg/mL puromycin as previously described [28]. PGK-nlacZ-MAR contains a phosphoglycerate kinase 1 promoter-driven nuclear localization signal (NLS)-lacZ and a chicken lysozyme matrix attachment region (MAR) to confer a high level of site-independent expression [29]. Low serum medium consisting of Dulbecco's Modified Eagle's Medium (DMEM) supplemented with 5% horse serum was used for myogenic differentiation assays.

### Plasmid Vector Construction

pCS2-EF-MyoD, a lentiviral vector carrying an EF1 α-promoter-driven mouse *MyoD* gene, was used for MyoD over-expression experiments as previously described [29]. Lentivirus-derived shRNA vectors for MyoD-knockdown (KD) were created as previously described [30]. For MyoD-KD experiments, culture supernatant from 293T cells transfected with different lentiviral vectors was used for infection of myoblasts. MyoD expression was assessed by RT-PCR, immunohistochemistry and western blotting. pMX-lacZ, a retroviral vector carrying a CMV promoter-driven lacZ gene, was used for bEnd co-culture experiments [77]. For retroviral vector production, pMX-lacZ vectors were transfected into Plat-E cells using Lipofectamine 2000 (Invitrogen). LacZ expression was assessed by 5-bromo-4-chloro-3-indolyl-beta-D-galactopyranoside (X-gal, Invitrogen) staining.

### Myocardial Infarction (MI) and Cell Transplantation

MI was induced by left coronary artery ligation as previously described [78]. Briefly, the left coronary artery was permanently ligated using a 9–0 nylon surgical suture, resulting in an infarct size equivalent to 30% of the mass of the LV. Sham operations were performed by following the same protocol but omitting the ligation step. Immediately after the left coronary artery ligation, viable mice were randomized into the following 3 groups: (1) *MyoD^−/−^* myoblast recipients; (2) wild-type myoblast recipients; and (3) medium only recipients. For the *MyoD^−/−^* and wild-type myoblast groups, 1×10^6^ cells were re-suspended in DMEM supplemented with 2% FBS and injected directly into the peri-infarct region of the myocardium 3–4 times using a 32-gauge needle. After injection, the mice were allowed a recovery period ranging from 1 day to 4 weeks.

### Echocardiographic Studies and Measurement of MI Size

Mice were lightly anesthetized with ketamine HCl (50 mg/kg, IP) and xylazine (16.5 mg/kg, IP). Echocardiography was performed using a SONOS 5500 echocardiographic system equipped with a 15.6-MHz phased-array transducer (Phillips, Netherlands). A two-dimensional short-axis view of the LV was obtained at the level of the papillary muscles. The LV internal dimensions, including end-diastolic dimensions (LVDd) and end-systolic dimensions (LVDs), were measured by the leading-edge method using a minimum of 8 consecutive cardiac cycles on the short-axis view of the LV. Ejection fraction (EF) was calculated using the following formula: EF = (LVDd^2^−LVDs^2^)/LVDd^2^×100. Following echocardiography, mice were euthanized and their hearts removed. MI size is expressed as a percentage of LV surface area as previously described [78].

### Evaluation of Engrafted Cell Number in the Infarcted Heart

Whole heart samples were stained with X-gal (Invitrogen) overnight as previously described [29]. Following X-gal staining, hearts were embedded in Tissue-Tek OCT compound (Fisher Scientific) and frozen in liquid nitrogen-cooled isopentane. A cryostat was used to produce 8 µm transverse tissue sections. Cell nuclei were counter-stained with 4′,6-diamidino-2-phenylindole (DAPI, Sigma-Aldrich). The number of engrafted cells was determined by examining 10 serial sections of whole heart for X-gal and DAPI double positive nuclei.

### Immunostaining

Eight µm sections of heart were fixed with 2% paraformaldehyde and incubated with primary antibodies followed by secondary antibodies. The following primary antibodies were used: anti-MEF2 antibody (Santa Cruz), anti-cardiac troponin T (Neo Markers), anti-laminin (Sigma-Aldrich), anti-nestin (Developmental Biology Hybridoma Bank), anti-caspase-3 (Abcam), anti-phospho-histone-3 (Millipore), anti-CD31 (BD Pharmingen), anti-Pax7 (R&D Systems), anti-MyoD (Santa Cruz), and anti-sarcomeric MHC (Developmental Hybridoma Bank). The following secondary antibodies were used: Alexa-488-conjugated anti-mouse IgG, Alexa-488-conjugated anti-rat IgG, Alexa-488-conjugated anti-rabbit IgG, Alexa-488-conjugated anti-goat IgG, Alexa-594-conjugated anti-mouse IgG, and Alexa-594-conjugated anti-rabbit IgG (all from Molecular Probes). Nuclei were counter-stained using DAPI. Microscopic images were captured by a DP-1 digital camera attached to BX51 fluorescence microscope with UPlanFLN objectives (all from Olympus). Photoshop CS2 (Adobe Systems) was used for image processing.

### Measurement of Vascular Density

Vascular density was measured by anti-CD31 antibody (BD Pharmingen) staining for 3 to 5 randomly selected non-overlapping fields in each cross section of infarct scar area of heart 1 week after myoblast injection, or tibialis anterior (TA) muscle of wild-type and *MyoD^−/−^* mice. The vascular density was calculated as the total number of CD31^+^ capillaries per section as previously described [79].

### Fluorescent Activated Cell Sorting (FACS)

Dissociated cells were prepared from the border and infarct areas of damaged heart and from sham mouse heart after digestion with collagenase type B and dispase II (Roche Diagnostics) [30], [76]. FACS analysis was performed using a FACS Calibur (BD Biosciences) equipped with double lasers. The following antibodies were used: allophycocyanin (APC)-labeled anti-CD31, phycoerythrin (PE)-labeled anti-Sca-1, and fluorescein isothiocyanate (FITC)-labeled anti-CD45 (all from BD Pharmingen). APC-labeled anti-rat IgG, PE-labeled anti-rat IgG, and FITC-labeled anti-rat IgG were used for control experiments (all from BD Pharmingen). Alexa 488 and PE were excited by a 488 nm argon laser and detected using FL1 (530/30) and an FL2 (576/26) filters, respectively. APC was excited by a 633 nm red diode laser and detected using an FL4 filter (620/20). Gates were strictly defined based on single antibody-stained control cells as well as the forward scatter (FSC) and side scatter (SSC) patterns.

### Endothelial Cell Culture

C3H10T1/2 fibroblast cells (10T1/2 cells) and brain-derived endothelial cells (bEnd cells) were obtained from the American Type Culture Collection (ATCC) and maintained in DMEM supplemented with 10% FBS. For co-culture experiments, bEnd cells were maintained with equal numbers of 10T1/2 cells, wild-type myoblasts, or *MyoD^−/−^* myoblasts in myoblast growth medium. To stimulate angiogenesis, bEnd cells were cultured in DMEM supplemented with 10% FBS and 100 ng/ml CX3CL1 (GenWay Biotech), 100 ng/ml FGF7 (GenWay Biotech), 100 ng/ml BDNF (Shenandoah Biotechnology), 100 ng/ml VEGF-D (Antigenix America), 100 ng/ml SDF-1 (R&D Systems), or 100 ng/ml PlGF (Shenandoah Biotechnology). After 3 days of culture, cells were stained using an anti-VE-cadherin antibody (PharMingen), Vectastain Elite ABC Kit (Vector Laboratories), and 3-Amino-9-ethylcarbazole (AEC, Sigma-Aldrich). Endothelial cell proliferation was assessed by counting at least 100 VE-cadherin positive cells for each experiment.

### Hypoxic Cell Culture

Hypoxia was established in anaerobic culture jars using a GasPak system (Becton Dickinson) with a palladium catalyst capable of producing a 0.2% oxygen level within 24 hours as previously described [80]. bEnd cells co-cultured with 10T1/2 cells, wild-type or *MyoD^−/−^* myoblasts were maintained under normal and hypoxic conditions for 1–3 days. Viable cells were counted after Trypan Blue (Invitrogen) staining. Endothelial cell survival was calculated as the number of X-gal positive cells after bEnd cell infection with a pMX-lacZ vector. For hypoxic conditions, twice as many wild-type myoblasts were used because of their relatively low survival rate compared to *MyoD^−/−^* myoblasts (half the survival rate after 3 days of culture under hypoxic conditions). Myoblasts were detected using an anti-Myf5 antibody (Santa Cruz Biotechnologies) and a Vectastain Elite ABC Kit (Vector Laboratories).

### Microarray Data Analysis

The microarray data used in this study was previously produced and described [29]. Briefly, total RNA was isolated from 2 independently prepared low passage (passages 6–8) myoblast cultures (wild-type; n = 2, *MyoD^−/−^*; n = 2) by acid-phenol extraction. Mu211K Affymetrix arrays contain approximately 13,000 genes and expressed sequence tags (ESTs). Data analysis was performed using GeneSpring 3.2.2 software (Silicon Genetics, Redwood City, CA) as previously described [29]. Each *MyoD^−/−^* chip (n = 2) was compared to a wild-type chip (n = 2) to examine disparities in gene expression. Difference calls demonstrating reproducible results in average comparisons were extracted for further analysis.

### Semi-quantitative RT-PCR

Total RNA was isolated from cells and tissues using TRIZOL (Invitrogen). Purified RNA was reverse-transcribed (Roche: Transcriptor First Strand cDNA Synthesis Kit) followed by 20–35 PCR cycles (Eppendorf Thermal Cycler) using the gene specific primer pairs described in Table 1. Optimal PCR cycles for each pair were determined by several different amplifications of the PCR products. Quantitative analysis was performed using Image-J software from the NIH. Relative expression was calculated using an internal β-actin control.

### Statistics

All data are expressed as mean ± SEM. For transplantation experiments, differences among groups were compared by a two-tailed Student’s t-test with a two-sample equal variance. A repeated measures two-way analysis of variance (ANOVA) and Bonferroni post-hoc measures were used to determine differences in cardiac function, angiogenesis and cell number between control and experimental groups with time as the repeated factor. Asterisks or double asterisks indicate experimental pairs where differences between the compared values were statistically significant (*p*<0.05) or (*p*<0.01), respectively.
